# Poor-Law Infirmaries and General Hospitals

**Published:** 1887-01-29

**Authors:** 


### POOR-LAW INFIRMARIES AND GENERAL HOSPITALS.

### DISCUSSION ON COLONEL MONTEFIORE'S PAPER.

THE following is a condensed account of the discussion which followed the reading of Colonel Montefiore's paper, published last week in THE HOSPITAL:—

DR. STEELE, the superintendent of Guy's, remarked that Poor-law Infirmaries were administered at a much lower rate than the Metropolitan Hospitals. In the General Hospitals, one patient cost not much less than one hundred pounds a year. The expenditure in Infirmaries fluctuated with the number of patients. A thousand beds cost £20 each, and the smaller ones according to the number in the infirmary. In our large General Hospitals, the cost of each patient per annum was nearly £100; and at Guy's, a little over £80. This included the out-patient expenditure as well as that of the household. These sums varied according to the way the patients were treated. At some hospitals the patients were not provided with some of the necessaries of life; friends had to supply such things as tea, sugar, and butter. All these things must be taken into consideration, when we compared one institution with another. The nursing in some of the infirmaries was much better than in others, but the nursing was, as a rule, much the same as it was fifty years ago in the General Hospitals. The General Hospitals had much improved their nursing. The hospital staff was now almost as expensive a matter as keeping the patients. As far as malt liquor was concerned, the nurses and staff at Guy's consumed double what the patients did. Beer was looked upon as an article of food, and he thought the working classes were all the better for their regular dose of beer per day. People actively employed, required it to support nature. The patients of course, had milk, which cost about the same as beer. The most interesting part of the paper they had just heard, was, he thought, that referring to the utilisation of the workhouses by students, for the study of medicine. This subject had been before them some time. He had never yet given his own opinion, but he felt strongly upon the question. Formerly, before infirmary wards were added to the Unions, the sick poor used to be largely sent to the hospitals for treatment. The parishes got into the habit of sending their cases. The charge made by the hospitals gradually grew from sixpence a day to two shillings. His hospital, for example, once drew £400 or £500 a year in this way. Since the Unions had established infirmaries, very few such cases now came to the hospitals. He thought such a system was serviceable alike to the Union and the hospital. They were most interesting cases, selected as fit subjects of treatment. Several hospitals had now wards shut up. He saw no reason why they should not be occupied by such cases as these.

DR. LLOYD (Lambeth Infirmary) endorsed what Colonel Montefiore had said with regard to the workhouses of the past. The State had recognised its duty to the paupers, and the work was done in the proper way at present. Infirmaries were not voluntary institutions; they were rate-supported. The question of circular wards was an exceedingly interesting one, but, so far, they did not know very much how the experiment was going on. The nursing of these infirmaries was a very important matter. There was only one infirmary in London which had the advantage of being able to start fairly in its nursing. At his own institution, they had to train their nurses as they went on. The question of cost had been largely entered upon, and an attempt had been made to compare the Poor-law Infirmaries with the General Hospitals. The two things were not parallel at all. As to the reception of pauper cases at the hospitals, he was quite certain that the General Hospital would not admit the class of cases which the infirmaries would send. He thought the duties of Guardians should be taken up by persons of better social position, and that it should not be left to petty shopkeepers to control the expenditure.

DR. G. W. POTTER observed that the principal point which arose in the paper was whether the wards of the Infirmaries should or should not be opened for clinical teaching. His own experience was that, after medical men had obtained their degrees they had a very great deal to learn. That ought not to be so. They had learnt physiology, anatomy, etc., to some extent; but what a medical man wanted was to know something of the details of practice in common cases. They all wanted to study "interesting cases" no doubt, but they wanted to study common cases as well, and he thought they should have the Poor-law Infirmaries open for such study. For instance, he had never come across a case of aneurism of the aorta in an experience of ten years. If students or young practitioners had the Poor-law Infirmaries open so that they could go there and devote a few hours a day for three or four days a week, they would know better what to do for their patients. Three-fourths of the younger doctors did not know, as they ought to know, the practical part of their profession.

Mr. Burdett, dealing with the subjects of nursing and clinical instruction, remarked that it had been said—and it had been felt—that the cost of good nursing was so great that the Poor-law Infirmaries could not provide it for their inmates. What the State undertook, the State was bound to do properly, and the ratepayers should hold the Guardians responsible for anything that went wrong. He maintained that at some of the Infirmaries, there were very grave abuses existing to this day. Those things were largely due to imperfect and improper nursing. It was a part of the hospitals' work to have an efficient nursing home, and the material within the hospital should be utilised for the public good, not only for training medical men, but nurses also, to go out and nurse the people. He was certain there were enough women who wanted to learn nursing. In this connection he would say, from his correspondence with America, that there was a wide field there for English nurses at a very high rate of wage, and they would be gladly welcomed if they could find their way across the Atlantic. Coming to clinical teaching, he canvassed the argument put into the mouth of an average guardian, to the effect that he, the guardian, would not permit medical teaching in the Infirmaries. If he were seriously ill, he would, for one, be quite willing that he should be a clinical case for the time being, and any good that could be got out of him as a case, was quite at the disposal of the students on the occasion he had been seriously ill in a hospital. To say that it was a wrong, an injustice, and a cruelty to ask the pauper to submit himself to clinical treatment was, in his opinion, monstrous. What if the patient said, "I object to being examined"? Why, some of the paupers objected to have a bath. If they had clinical teaching in the Infirmaries it would be immensely healthy to them; it would introduce a beneficial interest by making life a reality there, instead of the present dead level of monotony, which was one of the most dreadful things that all the Infirmary inmates had to face. The pay patients in America submitted to clinical teaching. They were put into the same wards as the free patients, and they liked it. He saw no reason why the nursing should not be made absolutely efficient in the Poor-law Infirmary, or why clinical instruction should not be made general. The Guardians would then begin to realise what the hospitals have realised, that it was better for every one that they should have touch with the outside public. He thought that they ought to thank Colonel Montefiore for his paper, because it showed that he had devoted a great amount of time to his subject.

Mr. Lunn said that at his Infirmary (Kensington), they had one of the best Boards, but unfortunately they could get no new blood. Such gentlemen would not come forward. When he sounded his Board on the question of clinical treatment, they would say that the paupers were not there for experiment, or some such argument as that. All felt that there was a great deal of material in our large infirmaries, if only something could be done towards aiding the medical profession to avail themselves of it. After referring to the old Poor-law Nurses, Mr. Lunn said that the Infirmary poor required good nursing as much as hospital cases. At Kensington they had one nurse to sixteen patients. He thought that the expenditure of infirmaries would compare well with that of hospitals. Hospitals required to be looked into as well as infirmaries.

Mr. P. Michelli, Secretary of St. Mary's, Paddington, defended the hospitals. One reason why the Poor-law Infirmaries were so cheap as compared with hospitals, was due to the difference of nursing. It had just been said that at Kensington Infirmary they had one nurse to sixteen patients. At his hospital they had one to five. In others, it was one to six or seven. Moreover, you could not have students about hospitals without increasing the expenses.

Mr. Pearce, of the Paddington Children's Hospital, said that at voluntary hospitals the surgical treatment and dressings were more expensive than at Poor-law Infirmaries.

The Chairman (Mr. Timothy Holmes, F.R.C.S.) summed up. The Paper, he said, had been an exceedingly interesting one, and one from which a great deal of instruction was to be derived. He had come there that evening as an old medical man to hear what suggestions could be made for the improvement of medical education. What Dr. Potter had said was exceedingly true. A large proportion of medical students obtained their legal qualification, but yet had a great deal to learn. The deficiency in their medical education was due to the fact that they saw too little of actual living patients. There was very much too little clinical teaching in London, and yet London contrasted well with many famous schools of medicine. It was painful to think that there were some 12,000 odd cases, such as they might meet with in ordinary practice, and yet that none of those cases could be made available for students. He was very disappointed to hear that such very feeble efforts had been made by the medical superintendents of these infirmaries to bring them into connection with the schools of clinical teaching. If the medical superintendents could move the Local Government Board to afford facilities of that kind, it would be a great step towards the improvement of medical education. There were differences with the Guardians, differences with the patients; but he must say he had never seen that patients in hospitals were inconvenienced by clinical teaching. It was an unmixed benefit to the man and to the hospital. He did not agree with Dr. Steele in his views as to the transfer of pauper cases to the hospital. The bulk of such cases were chronic cases.

